# Perspectives on the Orthopaedic Surgery Residency Application Process During the COVID-19 Pandemic

**DOI:** 10.5435/JAAOSGlobal-D-21-00091

**Published:** 2021-10-04

**Authors:** Amr M. Tawfik, Casey Imbergamo, Vivian Chen, Peter Filtes, Andrew Butler, Charles Gatt, Brian M. Katt

**Affiliations:** From the Rutgers Robert Wood Johnson Medical School, New Brunswick, New Jersey.

## Abstract

The COVID-19 pandemic has led to the cancellation of away rotations and other experiences fundamental to orthopaedic surgery residency application. Limited information is available on the experiences of applicants and program directors (PDs) during the COVID-19 pandemic residency application cycle. This study aimed to evaluate the current perspectives of applicants and PDs regarding the orthopaedic surgery residency application process.

**Methods:**

This was a cross-sectional study using 2 independent but similar surveys that addressed multiple aspects of the application process during the COVID-19 pandemic, including perceived effect of virtual experiences. Between February and March 2021, the surveys were distributed to orthopaedic surgery applicants and orthopaedic residency PDs.

**Results:**

In total, 113 applicants (20.1%) and 29 PDs (19.6%) completed the survey. Applicants applied to 97.6 programs and received 13.3 interviews. They participated in 2.4 virtual away rotations. In total, 79.3% of programs reported offering some form of virtual opportunity, including virtual away rotations (24.0%), virtual happy hours (64.0%), and virtual conferences (64.0%). Programs offering virtual away rotations hosted 46.8 rotators and only invited back 54.5% for an interview. Applicants were most concerned about the lack of away rotations, the interview, and networking during this cycle, and 51% reported less confidence in matching. The most important factors for influencing applicant rank lists were perceived happiness of residents, resident camaraderie, and geographic location. However, residency program social events were not well replicated in a virtual setting.

**Discussion:**

The COVID-19 pandemic presented new challenges for applicants and PDs. Applicants had less clinical exposure and received less interview invites after virtual away rotations. Despite applying to more programs, applicants received fewer interviews than in previous years. The virtual experiences adopted in this cycle did not adequately replicate the social factors that applicants found most important when ranking a program. Even during the COVID-19 pandemic, PDs most highly valued away rotation performance, clinical rotation performance, and board examination scores when offering interviews.

Orthopaedic surgery continues to be one of the most sought-after medical residencies, requiring some of the highest United States Medical Licensing Examination (USMLE) step 1 scores, number of research experiences, and class rank of any specialty.^1,2^ Owing to the highly competitive nature of the applicants, there has been a growing importance on forging professional relationships and demonstrating clinical aptitude through participation in senior elective rotations, referred to as away rotations. Studies have shown that a strong performance on these away rotations is considered one of the most important criteria to successfully match into a residency program.^3,4^

However, in response to the COVID-19 pandemic, cancellation of away rotations, in-person interviews, and other experiences fundamental to the orthopaedic surgery application process were enforced by medical schools following a statement by the Association of American Medical Colleges.^5^ Orthopaedic residency programs across the United States supported these guidelines and canceled commitments to host in-person away rotations and interviews. The loss of these experiences introduced gaps in both program directors' (PD) assessment of applicants and applicants' evaluations of prospective programs.

To compensate for this, programs rushed to produce novel virtual experiences, such as virtual away rotations, interviews, and social experiences. However, implementation of such program experiences varied among institutions. Some offered highly structured experiences, including virtual grand rounds, didactics, and journal clubs, whereas others only offered virtual open house sessions. It is unclear how valuable these experiences have been for applicants and PDs, and if they have adequately replicated their in-person counterparts.

Previous studies have evaluated multiple aspects of the orthopaedic surgery residency application process.^2,6,7^ However, there is limited information regarding the experiences of applicants and PDs during the COVID-19 pandemic residency application cycle. The unique challenges posed in the current cycle and the constantly evolving medical landscape warrant assessment. The primary goal of this study was to evaluate the current perspectives of applicants and PDs regarding the orthopaedic surgery residency application process during the COVID-19 pandemic.

## Methods

This was a cross-sectional survey study of applicants and PDs in orthopaedic surgery. The applicant cohort included all fourth-year medical student applicants to our institution's orthopaedic surgery residency program. All applicant e-mails were acquired from the Electronic Residency Application Service. The PD cohort included all PDs of Accreditation Council for Graduate Medical Education–accredited orthopaedic surgery residency programs. All PD e-mails were acquired through the publicly available data on the Accreditation Council for Graduate Medical Education website.

We designed two related anonymous surveys intended to capture the perspectives of applicants and PDs on multiple aspects of the orthopaedic surgery residency application process. The full contents of the applicant and PD surveys are given in Appendix 1, http://links.lww.com/JG9/A161, and Appendix 2, http://links.lww.com/JG9/A162, respectively. Between February 21 and March 15, 2021, the anonymous surveys were distributed to our acquired e-mail lists. Applicants who completed the survey were offered a chance to win one of 10 $50 Amazon gift cards. E-mail reminders were sent to applicants and PDs who had not yet completed the survey for a total of three cycles scheduled 5 days apart. One final e-mail was sent to both applicants and PDs on March 13th with a modification to ask for their assistance in adequately powering the study. The survey was closed just before match results on March 15th to minimize respondent bias.

Survey responses were collected and analyzed using R Statistical Software (R Core Team, 2017). Continuous data were reported as mean and standard deviation, and categorical data were reported as percent of total group. Categorical Likert style response data were assigned numerical values (from 1 to 5) and reported as weighted averages. The Student *t*-test and chi-square test were used for statistical analysis of continuous and categorical variables, respectively. Significance was established at a *P* value of <0.05.

## Results

The survey was successfully sent to a total of 562 applicant and 148 PD e-mails. In total, 113 applicants (20.1% response rate) and 29 PDs (19.6% response rate) completed the survey.

### Applicants

Applicants who responded to the survey were 27.9 ± 3.0 years old, with 69.1% identifying as male and 30.0% identifying as female. The geographic distribution of applicant responses was 53.6% northeast, 20.9% south, 20.0% midwest, and 5.5% west. In total, 80% of respondents stated that their medical school had an associated orthopaedic residency program. The average step 1 score was 246.3 ± 15.0. Applicants spent an average of 10.2 ± 4.9 weeks on in-person orthopaedic services. They applied to 97.6 ± 34.7 programs and received interviews at 13.3 ± 9.1 programs. In total, 53.2% of applicants participated in virtual away rotations. This group participated in 2.4 ± 2.1 virtual away rotations and received interviews at 1.6 ± 1.5 programs where they performed a virtual away rotation.

Table 1 summarizes applicant perceptions of the residency application during the COVID-19 pandemic application cycle. The residency application components applicants were most concerned about during the 2021 application cycle were away rotations (77.7%), networking (53.4%), and the interview (45.6%). The reported “other” concern was overall grades. Half of applicants (50.9%) had less confidence in matching because of COVID-19 pandemic, and 39.1% reported no effect on their confidence. For most applicants, COVID-19 pandemic had a negative effect on exposure to orthopaedics, orthopaedic subspecialties, and procedures. COVID-19 pandemic was less likely to have a negative effect on applicants' ability to participate in academic research, with 52.7% reporting no effect and 20.9% reported a positive effect.

**Table 1:** Applicant Perceptions of Residency Application During the COVID-19 Pandemic

Are you more concerned about any of the following components of your residency application because of the COVID-19 pandemic?	
Research experience	22.3%
Board examinations	19.4%
Away rotations	77.7%
Performance on clinical rotations	9.7%
LOR	19.0%
Networking	53.4%
Leadership experience	0.0%
Volunteer/community service experience	2.9%
Interview	45.6%
Inclusion into AOA	18.4%
Personal statement	4.9%
Other	1.9%
Has COVID-19 Pandemic affected your confidence in matching in orthopaedics?	
Yes, more confidence in matching	10.0%
No, same confidence in matching	39.1%
Yes, less confidence in matching	50.9%
COVID-19 Pandemic had _____ effect on my exposure to orthopaedics	
a positive	5.5%
no	20.9%
a negative	73.6%
COVID-19 Pandemic had _____ effect on my exposure to orthopaedic subspecialties	
a positive	4.5%
no	27.3%
a negative	68.2%
COVID-19 Pandemic had _____ effect on my exposure to orthopaedic procedures	
a positive	3.6%
no	29.1%
a negative	67.3%
COVID-19 Pandemic had _____ effect on my ability to get LOR	
a positive	5.5%
no	64.5%
a negative	30.0%
COVID-19 Pandemic had _____ effect on my ability to interact with faculty and mentors	
a positive	7.3%
no	41.8%
a negative	50.9%
COVID-19 Pandemic had _____ effect on my ability to participate in academic research	
a positive	20.9%
no	52.7%
a negative	26.4%
Transition to online and virtual learning had _____ effect on my orthopaedic education	
a positive	14.5%
no	44.5%
a negative	40.9%

LOR = letters of recommendation.

Table 2 summarizes factors regarding the interview process and of applicants' ranking of residency programs. Most applicants (76.4%) would prefer traditional in-person interviews if given a choice. The lack of away rotations during this cycle affected the rank list order for 81.8% of applicants. The Pandemic led to 53.6% of applicants applying to more programs; however, 41.8% reported applying to the same number of programs as originally intended. The most influential factors for their rank list in descending order were perceived happiness/quality of the life of the current residents, resident camaraderie, geographic location, and their interview experience. Most applicants reported participating in program-specific virtual events outside of the interview; however, they did not play a large role in determining the rank list. “Other” virtual events noted were question and answer sessions, grand rounds, webinars, and weekly academics. The most impactful virtual interview experiences considered when creating a rank list were the faculty and resident interviews. Letters of intent to a program were sent by 66.1% of applicants.

**Table 2:** Applicant Perceptions of Interviewing and Program Ranking During the COVID-19 Pandemic

If you were to participate in interviews in a traditional year, what would you most prefer?	
Traditional in-person interviews	76.4%
Virtual interviews	13.6%
Centralized location for all interviews	2.7%
Regional interviews	5.5%
No preference	1.8%
Do you think the lack of in-person away rotations affected your rank list order?	
Yes	81.8%
No	18.2%
Did the COVID-19 pandemic result in your applying to a different number of programs than you originally intended?	
Yes, I applied to more	53.6%
No, I applied to the same number	41.8%
Yes, I applied to less	4.5%
How important were the following factors in influencing your rank list?^a^	
Perceived happiness/quality of life of the current residents	4.38 ± 0.86
Resident camaraderie	4.46 ± 0.83
Virtual away rotations	2.19 ± 1.15
Personal interaction (online or in-person) with a current resident	3.73 ± 0.99
Experience during interview	4.03 ± 0.90
Geographic location	4.07 ± 1.00
Case volume	3.53 ± 1.00
Perceived early surgical experience	3.41 ± 1.15
Successful placement in desired fellowship	3.86 ± 1.08
Advice by mentor or other trusted source	3.64 ± 1.00
National reputation of program	3.51 ± 1.01
Size of program	2.92 ± 0.98
Need or preference of spouse/significant other	2.87 ± 1.56
Perceived likelihood of matching the program	2.51 ± 1.24
Did you participate in any program-specific virtual events outside of the interview?	
Virtual away rotations	57.6%
Virtual happy hours	90.9%
Virtual conferences	61.6%
Other	7.1%
How influential were virtual away rotations in informing your rank list?^a^	2.78 ± 1.18
How influential were virtual happy hours in informing your rank list?^a^	2.35 ± 0.90
How influential were virtual conferences in informing your rank list?^a^	2.00 ± 0.86
How much did the following experiences during virtual interviews affect your rank list?^a^	
Preinterview/postinterview social/“dinner”	2.53 ± 1.32
Faculty interviews	3.73 ± 1.00
Resident interviews	3.68 ± 1.12
Facility tours	1.91 ± 1.34
Informational talks	2.57 ± 1.01
Did you send out a letter of intent (a letter notifying a program that you have placed them first on your rank list) to a program?	
Yes	66.1%
No	33.9%

a Respondents reported the data for this section categorically (from no effect = 1 to very large effect = 5 or not at all important = 1 to extremely important = 5). These responses were converted to numerical scores and presented as weighted averages.

### Program Directors

The geographic distribution of PD responses was 31.0% northeast, 31.0% midwest, 24.1% south, and 13.8% west. The average number of residents per year at these programs was 4.8 ± 3.3. This year, programs received an average of 530.6 ± 233.5 applications as compared with a typical year where they received 493.8 ± 229.4 applications (*P* = 0.550). They conducted 52.1 ± 31.5 interviews this year as compared with 52.0 ± 29.4 interviews in a typical year (*P* = 0.986). Programs required 2.8 ± 0.39 letters of recommendation (LOR) this year as compared with 2.9 ± 0.59 in a typical year (*P* = 0.412).

In a typical year, programs hosted 22.1 ± 18.7 away rotators and offered interviews to 79.9% of away rotators. On average, away rotators accounted for 51.3% of applicants who matched to a program. This year, 79.3% of programs reported offering some form of virtual opportunity, including virtual away rotations (24.0%), virtual happy hours (64.0%), virtual conferences (64.0%), and other (16.0%). The other category included virtual town halls, question and answer sessions, and videos about the program. Programs offering virtual away rotations hosted 46.8 ± 43.6 rotators and only invited back 54.5% rotators for an interview.

Table 3 summarizes PD perceptions of the residency application during the COVID-19 pandemic application cycle. Most PDs (82.8%) prefer in-person interviews. Like applicants, PDs thought that the most important factors when creating a rank list were the faculty and resident interviews. PDs generally did not find that the interview day was well replicated virtually, with the preinterview/postinterview social events being rated the lowest. In total, 93.1% of PDs stated that receiving a letter of intent did not affect their ranking as an applicant.

**Table 3:** Program Director Perceptions of Residency Application During the COVID-19 Pandemic

If you were to host interviews in a traditional year, what would you most prefer?	
Traditional in-person interviews	82.8%
Virtual interviews	10.3%
Centralized location for all interviews	3.4%
Regional interviews	0.0%
No preference	3.4%
How much did the following experiences during virtual interviews affect your rank list?^a^	
Preinterview/postinterview social/“dinner”	2.14 ± 1.25
Faculty interviews	4.38 ± 0.86
Resident interviews	4.10 ± 1.11
Directly contacting student's letter writers/home programs	2.25 ± 1.29
Do you agree that the following experiences can be effectively replicated in a virtual interview?^a^	
Preinterview/postinterview social/“dinner”	2.41 ± 1.27
Faculty interviews	3.24 ± 0.99
Resident interviews	3.34 ± 0.94
Facility tours	3.00 ± 1.25
Informational talks	2.79 ± 1.35
Understanding applicant “fit” to program	2.90 ± 1.14
Did receiving a letter of intent (a letter from an applicant stating they have placed you first on their rank list) affect your ranking of the applicant?	
Yes, affected ranking positively	6.9%
No, did not affect ranking	93.1%
Yes, affected ranking negatively	0.0%

a Respondents reported the data for this section categorically (from no effect = 1 to very large effect = 5). These responses were converted to numerical scores and presented as weighted averages.

### Comparison of Applicant and Program Director Perspectives

Table 4 summarizes the social factors that applicants and PDs found most important for receiving/offering an interview and compares the responses between the two groups. Applicants overvalued LORs, networking, personal connection to a faculty member, being a student at the residency's institution, and their medical school's reputation compared with program PDs. However, applicants undervalued performance at the program's away rotation by comparison.

**Table 4:** Comparison of Applicant and Program Director Perspectives on the Most Important Factors for Receiving/Offering a Residency Interview During the COVID-19 Pandemic Application Cycle

Overall medical school grades	3.55 ± 1.04	3.62 ± 0.94	0.982
Performance on medical school clinical rotations	4.10 ± 0.95	4.21 ± 0.74	0.753
Research experience	3.82 ± 1.02	3.69 ± 1.04	0.577
Board examination scores	4.17 ± 0.89	4.14 ± 0.92	0.969
Performance on away rotation	3.71 ± 1.31	4.31 ± 1.20	0.022^a^
Letters of recommendation	4.56 ± 0.71	4.03 ± 1.02	0.015^a^
Inclusion into AOA	3.12 ± 1.24	2.86 ± 1.22	0.657
Personal statement	3.05 ± 1.15	3.43 ± 1.10	0.256
Leadership experience	3.16 ± 1.05	3.35 ± 0.87	0.413
Volunteer/community service experience	2.74 ± 1.14	3.06 ± 1.13	0.385
Networking	4.20 ± 0.92	3.00 ± 1.00	<0.001^a^
Program is in the same state as applicant hometown	3.22 ± 1.35	2.45 ± 1.21	0.006^a^
Family/personal connection with a faculty member at the program	3.51 ± 1.46	2.38 ± 1.27	0.001^a^
Being a student at the residency's institution	4.09 ± 1.19	2.76 ± 1.33	<0.001^a^
Being an undergraduate at the residency's institution	2.45 ± 1.21	1.83 ± 0.97	0.165
Being a collegiate athlete	2.61 ± 1.16	2.24 ± 1.21	0.487
Being a high school varsity athlete	1.81 ± 1.02	1.97 ± 0.94	0.621
Applicant medical school's reputation	3.51 ± 1.01	2.66 ± 1.23	0.001^a^
Applicant undergraduate program's reputation	2.23 ± 1.00	2.17 ± 1.07	0.804

Respondents reported the data for this section categorically (from not at all important = 1 to extremely important = 5). These responses were converted to numerical scores and presented as weighted averages.

a A *P* value of <0.05 signified a significant difference.

## Discussion

COVID-19 pandemic has undeniably affected the orthopaedic surgery residency application process for both applicants and programs. The unique challenges posed by this cycle made many applicants feel less confident in matching. These fears were not without merit because applicants applied to more programs (97.6 versus 74.9) but received fewer interviews than in previous cycles (13.3 versus 17).^8,9^ In addition, applicants had 10.2 weeks of in-person orthopaedic clinical time compared with 15.5 weeks in previous years, which decreased exposure to the field and its procedures.^2^ Furthermore, the in-person exposure this year was limited to a single institution, compared with multiple institutions in previous years. This reduction in exposure can be attributed to the cancellation of away rotations this year. This is concerning because Huntington et al^10^ found that an applicant's impression of a program after the away rotation played a major role in determining their rank list.

With away rotations and interviews being transitioned to virtual settings, both applicants and PDs were forced to hastily adapt to the new challenges presented. Our results found that many programs attempted some form of virtual opportunity to fill this gap. However, none of these events or opportunities played a major role in influencing applicants' rank list. Virtual interviews similarly missed the mark, with applicants and PDs overwhelmingly preferring in-person interviews. The lack of in-person away rotations due to COVID-19 pandemic affected the rank list for 82% of applicants. Historically, away rotations have been critical for both applicants and PDs. For PDs, away rotations present an opportunity to evaluate applicants' on work ethic, team fit, and clinical aptitude.^4^ For applicants, away rotations typically allow them to showcase their skills, gain LORs, and network at training sites aside from their home institution.^4^ O'Donnell et al^2^ previously found that the most important variable for applicants when selecting away rotations was their desire to match that program. This desire was met in previous cycles, with 80% of rotating students receiving an interview invitation. However, this year, programs only extended interview invites to 50% of their virtual away rotators. It is likely that PDs did not feel that they could adequately evaluate students in the virtual setting, translating to fewer interview invites.

Multiple factors affect whether an applicant will receive an interview invite. Historically, factors most strongly correlated with increased interview invites were Alpha Omega Alpha (AOA) status, board scores, and research productivity.^8,11^ Although board scores and research experience were ranked highly by both applicants and PDs in our study, the AOA status was not ranked highly by either. Despite its lower perceived importance, the AOA status likely correlates with the number of interviews received because AOA induction considers the totality of academic achievement, research, education, leadership, humanism, and service; all considered to be the characteristics of a successful orthopaedic resident.^12^

Our survey also underscored differences between applicants and PDs regarding factors considered important in the residency application process. Applicants tended to overvalue having connections to a residency's institution, reputation of their medical school, and LORs. Evidence supporting most of these perceptions exists. Cox et al^13^ found a strong association between the location applicants matched for residency and where the applicants previously lived or attended school. In addition, Schrock et al^14^ found that a higher proportion of matched applicants come from top 40 NIH-funded schools. Although these studies are based on matched applicants, applicants may use this information when gauging the likelihood of receiving an interview. These associations may explain why applicants think connections and school reputation are important for receiving an interview. Furthermore, applicants underestimated the importance of performance on away rotations for receiving an interview. This may provide another explanation for why virtual away rotations resulted in less interview invites compared with previous cycles (55% versus 80%).

Applicants reported that the most important factors when forming their rank lists were perceived resident happiness and camaraderie. This finding is consistent with previous years because the 2019 National Residency Match Program applicant survey and a study by Huntington et al. found that students applying to orthopaedic surgery listed perceived happiness and resident camaraderie among the most important factors when creating their rank list.^10,15^ Before this year, away rotations allowed students to become immersed within the institution's culture for four weeks, providing first-hand insight into resident camaraderie and contentment with the program. In addition, for students who did not complete an away rotation at the institution, programs typically hosted resident social events during the interview day, allowing the applicants to spend time with residents in an informal setting. Because these programs transitioned virtually, it may have been more difficult for applicants to confidently evaluate these factors. Although most applicants reported participating in virtual events outside of the interview, they did not play a large role in informing their rank list. This is unsurprising because PDs found that the virtual preinterview/postinterview social events were the least well-replicated events. Our findings should be considered when developing future virtual program-applicant events. One suggestion noted by Kenigsberg et al^16^ was limiting virtual resident-applicant experiences to 3 to 4 person groups to foster more meaningful interactions.

A letter of intent is an unsolicited letter from an applicant to a residency program mentioning the intention to list the program first on their rank order list. Of applicants who completed our survey, 66% reported sending a letter of intent to the program that they ranked first. However, 93% of PDs reported that letters of intent did not affect their ranking of applicants. A recent survey by Grimm et al^17^ similarly reported that only 5% of PDs across various specialties altered applicant ranking after a letter of intent. The lack of merit for a letter of intent may be due to applicants notifying multiple programs that the program is being placed in the first ranking position. Grimm et al^17^ also supported this notion, reporting that 53% of PDs experienced at least one incident of applicants falsely claiming that they are ranking the program as their first choice yearly. National Residency Match Program states in their participation agreement “Both applicants and programs may express their interest in each other; however, they shall not solicit verbal or written statements implying a commitment.”^18^

If the virtual format persists, programs should consider pairing rotating students with resident mentors. Others have shown success adopting similar measures, allowing students to work alongside residents during telemedicine visits and putting together case presentations.^19^ We also agree with Kenigsberg et al^16^ that virtual experiences should limit the number of students paired with residents to allow for more meaningful interactions. This may allow students to better gauge the social aspects/personality of the program while allowing residents to adequately gauge if rotating students would be a good fit for the program. Although there is likely no way to perfectly recreate in-person social experiences virtually, they can still provide added insight into the daily proceedings and educational style of a residency program.

We are aware of a few limitations with our study. First, our study had a low response rate for both surveys. There may also be a risk for selection bias because the responses represent those of applicants of a single institution. However, our geographic breakdown reflected current national trends because most resident training occurs in the northeast.^20^ Furthermore, PDs only represent part of the selection committee and may not entirely reflect how applicants are ultimately evaluated. Finally, our survey captured information primarily regarding applicant and PD perspectives and may not have reflected how either of these groups acted.

In conclusion, the COVID-19 pandemic presented new challenges for applicants and PDs, forcing an early adoption of virtual experiences. However, these programs left much to be desired. Applicants had less clinical exposure and received fewer interview invites after virtual away rotations. Despite applying to more programs, applicants received fewer interviews than in previous years. The virtual experiences adopted this cycle did not adequately replicate the social factors that applicants found most important when ranking a program. Even during the COVID-19 pandemic, PDs most highly valued away rotation performance, clinical rotation performance, and board examination scores when offering interviews. The findings of our study should be considered when developing future virtual and in-person orthopaedic residency opportunities.

## Supplementary Material

SUPPLEMENTARY MATERIAL
